# Beyond the Surface: Unveiling Hidden Hurdles to Primary Biliary Cholangitis Care

**DOI:** 10.7759/cureus.64753

**Published:** 2024-07-17

**Authors:** Omar Shamaa, Abdul Ahmed, Lora Rupp, Sheri Trudeau, Stuart C Gordon

**Affiliations:** 1 Gastroenterology and Hepatology, Henry Ford Health System, Detroit, USA; 2 Internal Medicine, Northwestern Medicine McHenry Hospital, McHenry, USA; 3 Center for Health Policy and Health Services Research, Henry Ford Health System, Detroit, USA

**Keywords:** end-stage liver disease, untreated, ursodeoxycholic acid, primary biliary cholangitis, fold consortium

## Abstract

Introduction: Ursodeoxycholic acid (UDCA) slows disease progression among patients with primary biliary cholangitis (PBC), yet not all patients receive this standard-of-care medication. Our study aims to identify reasons why PBC patients did not receive the recommended UDCA treatment.

Methods: Using medical record data collected by the Fibrotic Liver Disease (FOLD) Consortium for 2006-2016, we identified PBC patients from a single site with no UDCA therapy record. Two independent reviewers used a structured data collection instrument to systematically confirm and record the reasons for the lack of treatment.

Results: Among 494 PBC patients (11% men and 13.2% Black patients) with a median follow-up of 5.2 years, 35 (7%) had never received UDCA (16% men and 24% Black patients). Of these, 18 (51%) had laboratory indications of PBC but were not formally diagnosed. Among the remaining 17 patients with recognized PBC, six were never offered UDCA, seven declined treatment, and four remained untreated despite being offered treatment. We did not find a statistically significant association between the lack of PBC diagnosis and treatment and patients’ age (p = 0.139), gender (p = 0.222), race (p = 0.081), or insurance coverage (p = 0.456), perhaps due to our small sample size.

Conclusions: Multiple factors influencing the lack of evaluation and treatment in PBC patients were identified at the provider and patient levels. The most common reasons included financial barriers, loss to follow-up, severe decompensated disease at diagnosis, and lack of referral to specialists for further evaluation. Future interventions targeting modifiable provider and patient barriers may improve rates and timeliness of PBC diagnosis and treatment.

## Introduction

Ursodeoxycholic acid (UDCA) is the first-line therapy for primary biliary cholangitis (PBC), a chronic autoimmune disorder affecting the small intrahepatic bile ducts [1]. It is a well-tolerated medication with a relatively minimal side-effect profile [2]. It has also been shown that UDCA therapy slows the progression of PBC and reduces the need for liver transplantation, the risk of hyperbilirubinemia, and other clinical complications [3].

Although UDCA treatment is the standard of care, not every PBC patient is offered this medication. The United States-based Fibrotic Liver Disease (FOLD) Consortium previously found that men were less likely to receive UDCA than women and that Black patients were less likely to receive treatment than White patients [4,5], even though Black patients benefit significantly more from UDCA. We used in-depth chart abstraction of data from a single tertiary care center within the FOLD Consortium to identify the reasons why some patients did not receive the standard of care for PBC therapy.

## Materials and methods

The FOLD Consortium has been described previously [5]. FOLD follows the guidelines of the United States Department of Health and Human Services for the protection of human subjects, and the study protocol was approved by the institutional review boards of each of the 11 participating sites. We previously developed and validated an automated Classification and Regression Tree method [6] for accurately identifying PBC patients ≥ 18 years old who received care at the Henry Ford Health System between 2003 and 2014. Classifier variables included the following: (1) an antimitochondrial antibody (AMA)-positive test with at least one PBC diagnosis code; (2) an AMA-positive test with a recorded alkaline phosphatase (ALP) level > 150 IU/L (in the absence of a PBC diagnosis code); or (3) the presence of two PBC diagnosis codes (in the absence of an AMA-positive result). This classification model was validated using eight independent FOLD data sets and had an area under the receiver operator characteristic curve value of more than 93%.

PBC patients from a single FOLD site, Henry Ford Health, Detroit, Michigan, were further classified as having ever or never received UDCA. Henry Ford Health’s Institutional Review Board approval was obtained for the review of patient’s charts. Informed consent was waived because of the retrospective nature of the study (approval reference number: 9762). Two reviewers (OS and AA) independently performed manual chart reviews; for patients with no record of PBC therapy, they used a structured data collection instrument to systematically collect reasons why these patients never initiated treatment. Discrepant results between the two reviewers were discussed and resolved, or failing resolution, were adjudicated by the senior hepatologist (SG). During the chart review, patients who did not clearly meet the accepted American Association for the Study of Liver Diseases (AASLD) diagnosis criteria for PBC were further reviewed by the senior hepatologist for possible exclusion. The most updated AASLD diagnostic guidelines recommend that the diagnosis of PBC is generally based on the presence of at least two of the following criteria: (a) biochemical evidence of cholestasis with elevation of ALP activity; (b) presence of AMA; and (c) histopathologic evidence of nonsuppurative cholangitis and destruction of small or medium-sized bile ducts if a biopsy is performed. In the absence of histological evidence of PBC, AMA-positive patients with intermittently or mildly elevated ALP were included if there was no other cause to which the elevated ALP could be attributed.

Statistical analysis was performed using SAS, version 9.4 (SAS Institute Inc., Cary, North Carolina). Chi-squared tests were used to evaluate differences between PBC patients who did and did not receive UDCA therapy.

## Results

**Figure 1:**
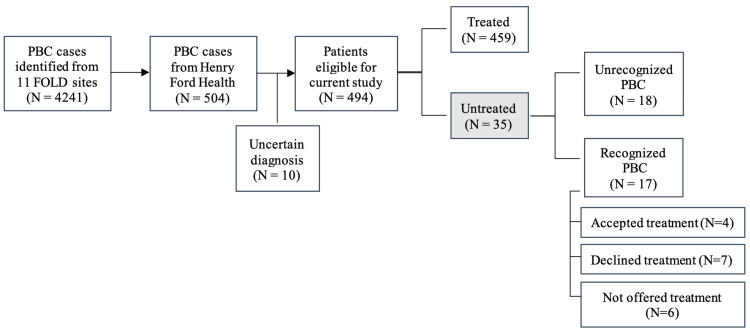
Flowchart of the study selection PBC: primary biliary cholangitis; FOLD: Fibrotic Liver Disease

Table 1 summarizes patient demographic and clinical characteristics by treatment status. Among the 504 patients who met the cohort inclusion criteria, 18 patients who did not clearly meet the accepted AASLD diagnosis criteria for PBC were further reviewed for possible exclusion. Of these, 10 were excluded because they did not meet the diagnostic criteria for PBC or there was insufficient evidence for a diagnosis (Figure 1) for a final sample of 494 confirmed PBC cases, with a median follow-up period of 5.2 years. Among the 494 confirmed PBC cases, 459 (93%) were appropriately diagnosed and treated, whereas 35 (7%) never received therapy.

**Table 1:** Characteristics of PBC patients by UDCA treatment status ASINPI: Asian + Pacific Islander + American Indian; PBC: primary biliary cholangitis; UDCA: ursodeoxycholic acid

Variable	Response	Treated	Untreated	P-value
		(N=459) N (%)	(N=35) N (%)	
Age, years	≤40	23 (5.01)	4 (11.43)	0.139
	40-50	83 (18.08)	2 (5.71)	
	50-60	157 (34.2)	13 (37.14)	
	>60	196 (42.7)	16 (45.71)	
Gender	Women	411 (89.54)	29 (82.86)	0.222
	Men	48 (10.46)	6 (17.14)	
Race	ASINPI/other/unknown	21 (4.71)	2 (5.71)	0.081
	Black	56 (12.56)	9 (25.71)	
	White	369 (82.74)	24 (68.57)	
Insurance	Medicaid	13 (2.83)	2 (5.71)	0.456
	Medicare Plus	181 (39.43)	11 (31.43)	
	Private	265 (57.73)	22 (62.86)	
Charlson-Deyo comorbidity score	0	144 (31.37)	11 (31.43)	0.037
	1	232 (50.54)	11 (31.43)	
	2	25 (5.45)	4 (11.43)	
	3	58 (12.64)	9 (25.71)	

Approximately half of the 35 untreated patients were diagnosed with PBC (17); of these, four patients accepted UDCA treatment, seven declined treatment, and six were never offered therapy by their provider. A variety of patient and physician factors contributed to the lack of diagnosis and treatment. The most common patient factors included mild symptoms, severe disease with rapid decompensation, active liver transplant evaluation, competing health concerns, loss to follow-up, and cost difficulties or lack of insurance coverage. Physician factors included lack of referral to a specialist by primary care providers and lack of specialist disease recognition, which resulted in reduced PBC diagnosis (Table 2).

**Table 2:** Patient and physician factors N/A: not applicable; PBC: primary biliary cholangitis; PCP: primary care provider

PBC Treatment Status (n)		Patient Factors (n)	Physician Factors (n)
Unrecognized PBC (18)		Severe disease/rapidly decompensating (3), transplant evaluation (3), loss to follow-up (5), asymptomatic/mild symptoms (1), competing health concerns (6)	Conflicting presentation (2), lack of diagnosis by a specialist (1), no referral to a specialist by PCP (6)
Recognized PBC (17)	Accepted (4)	Insurance/cost difficulty (3), competing health concerns (1)	None
	Refused (7)	Mild symptoms (3), severe disease (1), liver transplant (1), loss to follow-up (4), competing health concerns (2)	None
	Not offered (6)	Mild symptoms (4), severe disease (1), liver transplant (1), loss to follow-up (1)	N/A

Of the 17 diagnosed but untreated PBC cases in our cohort, six (35%) were never offered therapy because they were asymptomatic or had very mild symptoms at presentation. Among the seven patients who refused treatment, three (43%) had no or mild symptoms at diagnosis, favoring a wait-and-watch approach. Only two of the 17 diagnosed but untreated patients (12%) had severe liver decompensation: one refused treatment and was later denied liver transplantation due to cardiac risk factors; the second patient died within a week of PBC diagnosis before treatment was initiated. Eighteen of the 35 (51%) untreated patients in our cohort were never diagnosed with PBC. In contrast to the patients who had received a diagnosis but no treatment, four (22%) had rapidly decompensating severe liver disease: one patient died before a full PBC workup was initiated; the remaining three were evaluated for a liver transplant, but two died before receiving a transplant, and the third was delisted due to concurrent pancreatic cancer.

Three of the 18 (17%) patients with unrecognized PBC had competing health concerns: two had decompensated congestive heart failure and chronic obstructive pulmonary disease; the third patient suffered from terminal chronic lymphocytic leukemia with brain metastasis. Similarly, three of the 17 (18%) diagnosed PBC cases had competing health concerns that were the primary barrier to treatment: one had metastatic cancer with uncontrolled atrial fibrillation and died without initiating UDCA despite it being prescribed; the remaining two patients declined UDCA therapy due to metastatic lung cancer in one and end-stage renal disease in the other.

Among our 18 unrecognized PBC patients, five were lost to follow-up. In addition, of the 17 diagnosed but untreated PBC cases, four initially refused treatment and were later lost to follow-up, and one patient was lost to follow-up after the specialist was planning to repeat AMA titers and initiate treatment if they returned elevated.

Patients with a Charlson-Deyo comorbidity score of one were more likely to be treated than those with higher scores (p = 0.04). A more significant proportion of untreated patients were Black and men, although these differences were not statistically significant (p = 0.08 for race and p = 0.22 for gender). Forty-three percent (n = 194) of patients had Medicaid or Medicare insurance coverage, while 58% (n = 263) had private insurance. There was no statistically significant association between the lack of PBC treatment and patients’ age (p = 0.14) or insurance coverage (p = 0.46).

**Figure 2:**
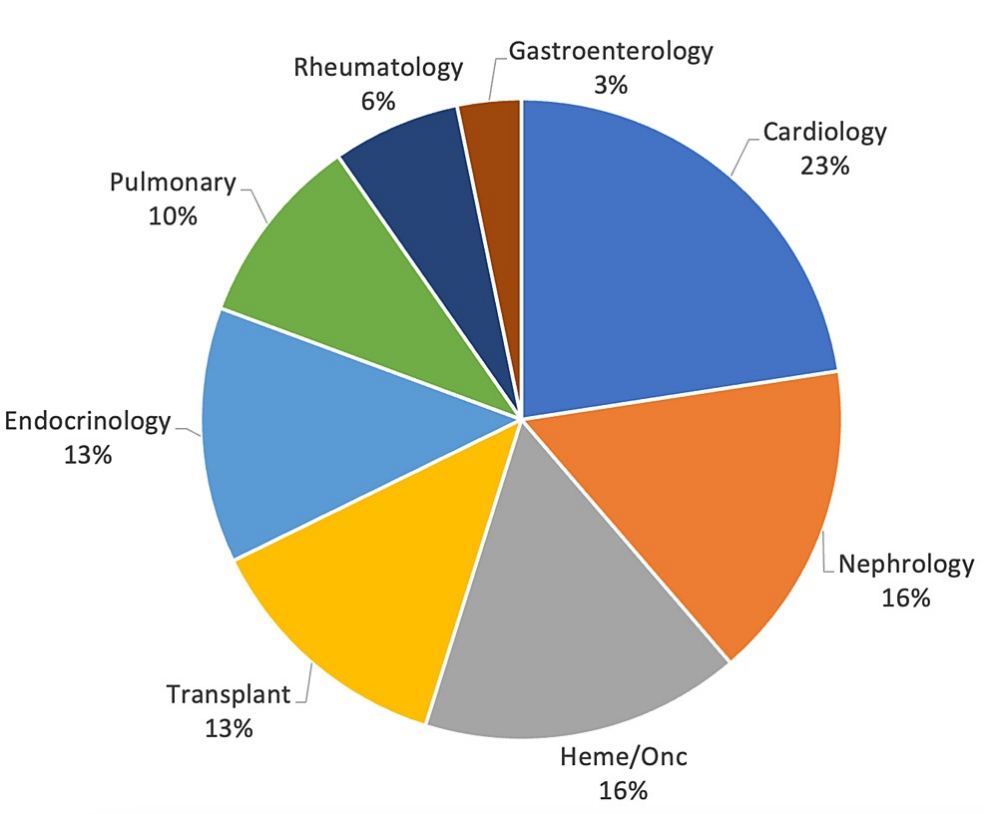
Specialties that followed patients with untreated PBC

**Figure 3:**
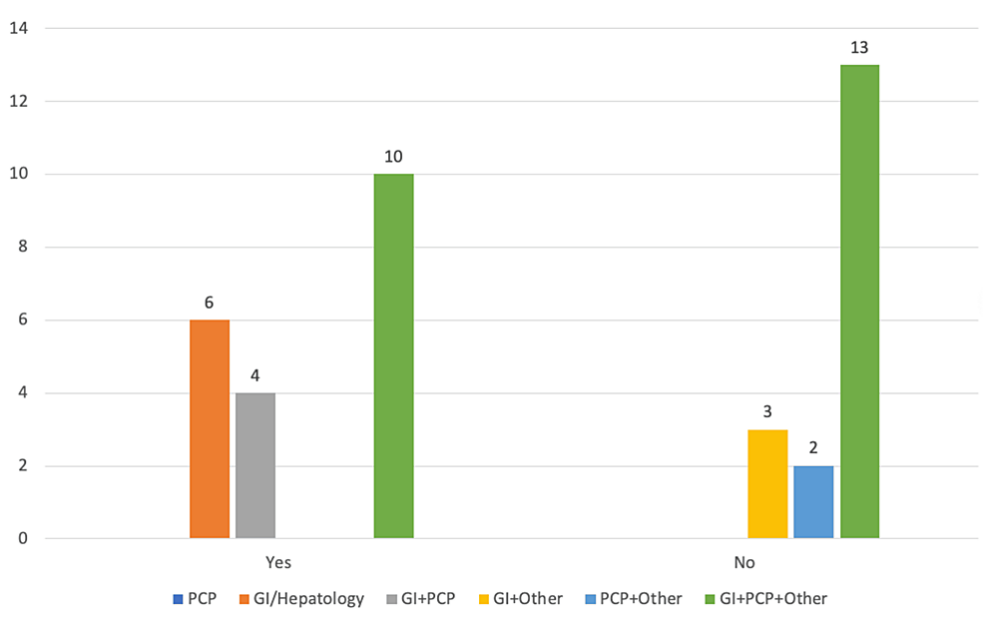
Do any Henry Ford Health providers recognize PBC diagnosis? GI: gastroenterology; PCP: primary care provider

Patients were seen by providers that included primary care, gastroenterology/hepatology, or another subspecialty, including cardiology (23%), nephrology (16%), hematology/oncology (16%), transplant (13%), endocrinology (13%), pulmonary (10%), and rheumatology (6%) (Figures 2, 3).

## Discussion

There is extensive literature describing the positive impact of UDCA on PBC symptoms and progression. Since its approval in 1997, UDCA has been clearly shown to delay progression to cirrhosis and increase survival times [3,7,8]. Epidemiologically, while PBC incidence has remained steady, prevalence has increased, likely reflecting earlier diagnosis and improved mortality [9]. However, some patients develop cirrhosis even with early initiation of UDCA [10]; therefore, all medical guidelines recommend lifelong follow-up to monitor variation in disease course [1,11,12]. If the first-line treatment fails, patients may also benefit from second-line treatment options such as obeticholic acid [13].

However, given the significantly shortened PBC survival time in the absence of adequate therapy, information regarding undiagnosed and untreated PBC patients is also critical [11]. Seven percent of our PBC sample never received therapy. Such patients are at risk for progressive liver disease; identifying and addressing barriers to early recognition and treatment can reduce disease burden and improve quality of life for patients with PBC.

Our analysis found no significant associations between patient characteristics and lack of PBC diagnosis or treatment (Table 1). This could be due to our small sample size. However, a trend toward significance regarding race was observed (p = 0.09); the proportion of untreated Black patients in our sample was approximately twice that of those who were treated. Previous studies have identified racial disparities in the diagnosis and treatment of PBC. For instance, Black patients with PBC were less likely to receive UDCA treatment than Whites, had higher liver transplant waitlist mortality, and had a higher mean model for end-stage liver disease scores at waitlist death [4].

The most common clinical factors among our untreated patients included mild symptoms or, in contrast, severe disease with rapid decompensation or active liver transplant evaluation. Additional patient-related factors included competing health concerns, loss to follow-up, and cost difficulties or lack of insurance coverage. Physician-related factors included lack of referral to a specialist by primary care providers, as well as specialist knowledge, attitudes, and information management that led to inadequate PBC recognition (Tables 2, 3).

**Table 3:** Summary of patient and physician factors with proposed interventions PBC: primary biliary cholangitis; PCP: primary care provider

Patient Factors		Physician Factors	
Factor	Intervention	Factor	Intervention
1) Mild symptoms	Patient education on disease course and treatment impact	Lack of diagnosis by non-specialist providers (PCP, others)	Educational interventions and use of electronic medical record health advisories for early identification of abnormal PBC laboratory tests
2) Severe disease/rapid decompensation	Prompt referral to transplant center	Lack of referral to specialists by primary care providers	Encouraging early referral to specialists, particularly in equivocal cases
3) Competing health concerns	Nursing aids to help arrange clinic visits, transport, and offer virtual visits	Specialist inadequate PBC recognition	Addressing provider gender and race disease bias
4) Loss to follow-up	Follow-up reminders via mail, email, or telephone	-	-
5) Insurance/cost difficulties	Social work referral for financial support	-	-

Studies have shown that the major costs associated with PBC are generally related to complications of cirrhosis and costs due to liver transplantation [14]. A recent study [15] showed that between 2005 and 2014, the average total charges for PBC increased by approximately 7%; however, costs have remained consistent after adjustment. Racial disparities in access to PBC also exist. For instance, fewer Hispanic patients with PBC had health insurance compared to non-Hispanic (86.5% vs. 98.1%; odds ratio 0.1, 95% CI: 0.0-0.9) [16]. Data regarding health insurance and outcomes among Black and ASINPI (Asian, Pacific Islander, American Indian) PBC patients are scarce; given that UDCA has been shown to significantly improve outcomes among those populations, a better understanding of how access to treatment impacts outcomes in these patients is vital. In our patient cohort, three (17.6%) of the recognized PBC patients accepted but were unable to start treatment due to lack of insurance coverage or inability to afford medication. Since most of the healthcare cost burden associated with PBC arises from lack of treatment, efforts to provide access to timely care and treatment may reduce disparities in addition to slowing disease progression and improving patients’ quality of life.

Early identification of PBC and initiation of treatment is crucial for slowing or preventing liver failure, transplant, or death. Among the 18 patients in our sample with unrecognized PBC, three of the 13 seen by a gastroenterologist or a hepatologist were never diagnosed due to the physician's lack of disease recognition.

The first of these patients was a 56-year-old female with persistently elevated liver function tests (> 15 years), including transaminases > 200 IU/L, who was first seen by gastroenterology in 1993. Testing showed a positive AMA (titer 1:320) and normal ALP level without other symptoms. No liver biopsy was performed, and the patient was never formally diagnosed or initiated on therapy, but liver disease did not progress further. The second patient was a 59-year-old male with cirrhosis complicated by hepatic encephalopathy, variceal bleeding, and ascites. Testing in 2010 showed a positive AMA (titer 1:320) and chronically elevated ALP that peaked at 438 IU/L. Alcohol was labeled as the primary etiology for the underlying liver dysfunction; PBC was also suspected, given the positive AMA and elevated ALP, but no liver biopsy was performed, and no official diagnosis was made. The patient was evaluated twice for a liver transplant but was not approved due to poor compliance and a history of cardiac disease; he died in 2015. The third patient was a 72-year-old female with a history of lung cancer. Her first positive AMA test was in 2016; ALP was persistently elevated (176-739 IU/L) but normalized in 2019. No liver biopsy was performed, and no formal diagnosis was established. The patient died from complications of lung cancer in 2022.

As seen with these patients, PBC can present with conflicting symptoms. Despite specific diagnostic criteria for PBC, some ambiguity remains that may delay diagnosis. Since PBC is most frequently diagnosed among women, White patients, and those 61-70 years of age [5], it may not be initially suspected among men and patients from other racial/ethnic groups. Another difficult-to-diagnose group includes patients who are asymptomatic or have non-congruent biochemical tests, specifically a positive AMA with normal transaminases and ALP. Importantly, however, a recent study of AMA-positive patients with normal ALP levels showed that most patients (68%) had liver histology indicating PBC. Forty-one percent were Ludwig stage three (severe fibrosis) or four (cirrhosis). Given these findings, liver biopsy should be considered if PBC is suspected, particularly if the ALP is within normal limits [17]. The absence of liver biopsies likely contributed to a lack of formal diagnosis and subsequent treatment in all three patients seen by our specialists.

Six of the 18 undiagnosed patients never received a referral for gastroenterology/hepatology evaluation from their primary care provider. Despite recent efforts to increase PBC awareness, our study highlights the need to further supplement PBC knowledge among primary care providers. For example, family practice providers, general practitioners, and dermatologists can identify itching as an early symptom of cholestasis due to PBC and initiate appropriate diagnostic testing. In addition, because many PBC patients may be evaluated outside of large specialty liver centers, information sharing through electronic medical record templates and “best practice” health advisories could flag patients’ laboratory results (e.g., a positive AMA test) that merit further assessment. Future studies should then assess the effectiveness of such novel strategies post-implementation and explore other interventions to optimize PBC diagnosis and treatment rates.

There are some limitations to the study. Some PBC patients had missing data; this lack of laboratory test results may have resulted in limited awareness of PBC among primary care physicians to prompt a referral and a lack of data points for specialty services to confirm a diagnosis. In addition, our sample size is relatively small since only one center was involved. However, given the small proportion of untreated PBC patients in the community, the findings of this study may be generalizable and offer new practical information for this patient population.

## Conclusions

Our study reveals that there are concerningly high proportions of probable PBC patients who have not received an appropriate diagnostic workup, resulting in a lack of treatment and increased risk for progressive liver disease. In addition, this study highlights the existence of multiple potentially modifiable factors at the patient and physician levels that lead to the lack of PBC evaluation and treatment. Future interventions targeting those barriers may improve the rates and timeliness of PBC diagnosis and treatment.
